# Predictive values of heart rate variability, deceleration and acceleration capacity of heart rate in post‐infarction patients with LVEF ≥35%

**DOI:** 10.1111/anec.12771

**Published:** 2020-07-07

**Authors:** Xiang Liu, Li Xiang, Guangming Tong

**Affiliations:** ^1^ Department of Cardiology The Second Affiliated Hospital of Soochow University Suzhou City China

**Keywords:** autonomic function, heart rate variability, myocardial infarction, sudden death

## Abstract

**Background and aims:**

The aim was to investigate the predictive values of heart rate variability, deceleration, and acceleration capacity of heart rate in sudden cardiac death in postinfarction patients with left ventricular ejection fraction (LVEF) ≥ 35%.

**Methods:**

We enrolled 138 acute myocardial infarction patients (MI) randomly in sinus rhythm with LVEF ≥ 35% after myocardial infarction. Data on heart rate variability, deceleration runs, deceleration, and acceleration capacity were obtained from 24h‐dynamic electrocardiogram recordings. Clinical characteristics, medications, and echocardiography data were noted. The endpoints were sudden cardiac arrhythmias (SCA), including malignant arrhythmias in the hospital and viewed sudden death out of the hospital. Relationships between autonomic parameters and endpoints were evaluated.

**Results:**

During follow‐up for over 24 months in MI patients, 10 patients occurred sudden cardiac arrhythmias. Subjects with SCA showed lower levels of SDNN (*p *= .018), TP (*p *= .007), VLF (*p < *.001), DC (*p < *.001), and low‐risk DRs (*p < *.001) than those without SCA. A low SDNN level (HR: 8.888, *p *= .006), low VLF level (HR: 14.699, *p *= .016), low DC level (HR: 4.430, *p *= .045), and higher risk DRs (HR: 3.81, *p *= .040) were identified as independent risk factors of SCA for postinfarction patients with LVEF ≥ 35%. The area under the ROC curve (AUC) of SDNN, VLF, and DC for identification of SCA were, respectively, 0.724 (*p *= .019), 0.807 (*p < *.001), and 0.804 (*p *= .002). SDNN, VLF, and DC combined assessment area under the ROC curve were 0.828 (*p < *.001).

**Conclusion:**

Decreased SDNN, VLF, DC, and abnormal DRs are independently associated with increased risks of sudden cardiac arrhythmias in post‐MI patients with LVEF ≥ 35%. Combined SDNN, VLF, and DC may help identify a high‐risk group of malignant arrhythmias in postinfarction patients.

## 1 INTRODUCTION

Sudden cardiac death is frequently encountered in postinfarction patients. It is a major clinical question in the USA (Mozaffarian et al., 2016), and the socioeconomic, as well as family burdens of cardiac death after myocardial infarction, is immense. Many cardiac patients who die from sudden cardiac death do not have left ventricular performance particularly compromised (Aro, Reinier, & Rusinaru, 2017), which proposed the urgent need to identify postinfarction patients at a higher risk. Time and frequency domain of heart rate variability measured by standard deviation of all normal RR intervals (SDNN), very low frequency (VLF, 0.005–0.04Hz), low frequency (LF, 0.04–0.15 Hz), and high frequency (HF, 0.15–0.40 Hz) bands were previously confirmed to be associated with increased risk of mortality in AMI patients (Bigger et al., 1992; La Rovere, Bigger, & Marcus, 1998). Assessment of deceleration and acceleration capacity of heart rate by the method of phase‐rectified signal averaging algorithm (PRSA) was first brought up by Bauer et al. Deceleration capacity was found a strong predictor of mortality after myocardial infarction, even better than left ventricular ejection fraction (LVEF) and conventional measures of heart rate variability (Bauer, Kantelhardt, & Barthel, 2006). At present, different proposals based on electrocardiography and combined autonomic markers, such as HR variability, heart rate turbulence, and deceleration capacity are made to identify special subjects at high risks. The combination of severely impaired baroreflex function with abnormal autonomic tone would improve risk prediction (Bauer, Barthel, & Schneider, 2009; Hamm, Stulpnagel, & Vdovin, 2017; Quintana, Storck, & Lindblad, 1997). In the present study, we investigated changes of autonomic parameters and their values as risk predictors for sudden cardiac death in postinfarction patients with LVEF ≥ 35%.

## 2 METHODS

### 2.1 Participants and clinical characteristics

Between March 2012 and October 2018, patients of acute myocardial infarction in sinus rhythm, and underwent percutaneous coronary intervention (PCI) admitted into the hospital for emergency treatment were enrolled in the prospective study. Myocardial infarction was diagnosed clinically as commonly described before (Bauer, Barthel, & Schneider, 2009): if a patient had at least two of the following findings: chest pain for ≥20 min, creatine kinase‐MB above the doubled upper normal limit of our laboratory, and ST‐segment elevation of ≥0.1 mV in two or more limb leads or ≥0.2 mV in two or more continuous precordial leads at the time of admission. Exclusive criteria were as below: left ventricular ejection fraction (LVEF) < 35% by Simpson echocardiography in hospital, cardiomyopathy, valvular heart disease, unrecovered complete atrioventricular block, and bundle branch block after PCI, life‐threatening bleeding, neoplasm, and rejected to participate in the study.

General characteristics, past histories such as diabetes, hypertension, and dyslipidemia were recorded. Dyslipidemia was diagnosed if a patient had at least one of the following findings: triglyceride ≥2.3 mM, low‐density lipoprotein ≥3.4 mM, total cholesterol ≥6.2 mM, or priorly diagnosed hyperlipemia. Fast blood samples for creatinine, lithic acid were drawn from the next morning admitted to the hospital and before discharge. eGFR was calculated by MDRD equation. Grace score and Gensini score were evaluated (Gensini, 1983).

### 2.2 Electrocardiographic analysis and echocardiography data

Myocardial infarction (MI) patients received 24 h ambulatory electrocardiogram (Holter) via DMS300‐3A after PCI before discharge. Normal daily living activities were allowed during the ECG recordings. Holter was analyzed by experienced cardiologists. Artefactual beat labels were manually eliminated via DM Software CardioScan II (version 12.5.0078a). The time and frequency domain of HR variability (HRV), deceleration and acceleration capacity and DRs were automatically computed and recorded. Relative counts of deceleration runs of 1–10 RR intervals were divided into low‐, intermediate‐, and high‐risk groups as has been noted (Guzik, Piskorski, & Barthel, 2012). In the present study, we encoded deceleration runs as 0–2 for low to high risk. Echocardiography was performed, and data were recorded.

This clinical study protocol was approved by the Ethics Committee, and informed consent was obtained from all subjects.

### 2.3 Follow‐up

All patients we enrolled in the present study received dual antiplatelet, anticoagulants (heparin or LMWH), statin drugs, positive inotropic, and hemodynamic support if necessary in hospital. The usage of β‐blockers, ACEI/ARB, spironolactone, and necessary drugs was traced during follow‐up.

Postinfarction patients were checked at least 24 months. Patients were followed up by home‐visit, call consultations to patients or family members, outpatient records, and electronic medical records system, etc. Drug usage of ACEI/ARB, aspirin, and spironolactone was recorded during the follow‐up period. The main end point of this study was the combined end point of sudden cardiac arrhythmias (SCA) including ventricular fibrillation, sustained ventricular tachycardia with hemodynamic deterioration detected by ECG monitoring or ECG, and viewed sudden cardiac death out of hospital, verified from the hospital and from either the physicians or those who had witnessed the death. Sudden cardiac death was defined taking the following items as references: (a) witnessed death occurring within 60 min from the onset of new symptoms, unless other noncardiac causes were obvious; (b) unwitnessed death (<24 h) without preexisting, advanced circulatory diseases, or other causes of death; or (c) unsuccessful resuscitation.

We also recorded the information of a combined end point of major cardiovascular events (MACE) consisting of myocardial reinfarction, hospitalizations for heart failure, progressive coronary artery lesions indicating another stent implantation, or other cardiovascular death at the earliest time after discharge. The initial time of follow‐up was set as the day after percutaneous transluminal coronary intervention, and the end point of follow‐up was the first onset of SCA events, MACE events, or the end date of follow‐up (August 2019). The follow‐up time was calculated in months.

### 2.4 Statistical analysis

The data were analyzed by the software of SPSS23.0 and Stata/SE 14.0. Continuous variables are expressed as a median and inter‐quartile range. Qualitative data are presented as absolute numbers and percentages. Pearson's χ^2^ tests and Mann–Whitney *U* test were used for multivariate comparisons of continuous and categorical variables, respectively. The receiver operating characteristic curve (ROC) was performed to determine the diagnostic values of variables in identifying SCA. Because patients might die before developing SCA, we used the competing‐risks regression to determine risk factors and to evaluate survival curves and hazard ratios for arrhythmias. For all analyses, values of *P < *0.05 were considered statistically significant.

## 3 RESULTS

In the present study, 159 patients were screened and enrolled at the initial time of the study, and 21 (15.2%) cases lost contact during follow‐up. 138 consecutive acute myocardial infarction (MI) was finally included. During follow‐up in MI patients, 10 (7.2%) occurred sudden cardiac arrhythmias, and MACE occurred in 29 patients, with 18 (13.0%) cases of hospitalizations for heart failure, 3 (2.2%) cases of myocardial reinfarction, 1 (0.7%) cases of death for cardiac shock, 7 (5.1%) cases of progressive coronary lesions. Clinical characteristics, blood tests, echocardiography data, and medications were shown in Table 1. Patients with SCA were older (*p* = .013) and had a higher Grace score (*p* = .035). Other variables such as the Gensini score, echocardiography data, and medications showed no difference between SCA and non‐SCA (Table 1).

**Table 1:** Comparisons of clinical characteristics, blood tests, echocardiography data, and medications between SCA and non‐SCA

Variable	non‐SCA (*n* = 128)	SCA (*n* = 10)	*p*
Clinical characteristics			
Male, *n* (%)	109 (85.2)	7 (70)	Ns
Age, years	65 (57.2,74.7)	79.5 (68.5,82.2)	.013
Diabetes, *n* (%)	32 (25.0)	2 (20.0)	Ns
Hypertension, *n* (%)	81 (63.3)	5 (50.0)	Ns
Smoking, *n* (%)	86 (67.2)	3 (30.0)	.018
Dyslipidemia, *n* (%)	45 (35.4)	3 (30.0)	Ns
Gensini score	53.3 (36.2,82.9)	86.0 (50.7,99.4)	Ns
Grace score	137.2 (120.0,159.0)	176.1 (127.2,186.2)	.035
Blood tests			
eGFR, ml/min/1.73 m^2^	93.5 (72.0,109.0)	68.0 (32.5,99)	Ns
Uric acid, µM	347.0 (272.0,413.5)	392.0 (356.0,480.5)	Ns
Echocardiography			
LAD, mm	42.8 (38.0,46.7)	41.5 (39.9,45.9)	Ns
LVDd, mm	52.8 (49.0,55.8)	51.5 (50.6,55.9)	Ns
LVDs, mm	37 (31.0,41.7)	38.0 (34.5,41.7)	Ns
LVEF, %	55.0 (46.8,63.0)	46.6 (43.0,62.0)	Ns
Pulmonary arterial pressure, mmHg	30.0 (24.0,37.0)	37.1 (26.7,41.2)	Ns
Medications			
ACEI/ARB, *n* (%)	79 (62.2)	5 (50.0)	ns
β‐blockers, *n* (%)	98 (77.2)	6 (60.0)	ns
Asprin, *n* (%)	122 (96.1)	10 (100.0)	ns
Spironolactone, *n* (%)	24 (18.9)	0 (0.0)	ns

Abbreviations: ACEI/ARB, angiotensin converting enzyme inhibitor/angiotensin receptor antagonist; eGFR, estimated glomerular filtration rate; LAD, left atrial diameter; LVDd, left ventricular end‐diastolic dimension; LVDs, left ventricular end‐systolic dimension; LVEF, left ventricular ejection fraction.

Comparing autonomic data between patients with SCA and non‐SCA, we found that SDNN (*p* = .018), TP (*p* = .007), VLF (*p < *.001), DC (*p < *.001) and the proportion of low‐risk DRs (*p < *.001) showed statistical difference between the two groups, whereas other time and frequency domain indexes of HRV and AC lost significance (Table 2).

**Table 2:** Comparisons of autonomic parameters between SCA and non‐SCA group

变量	non‐SCA (*n* = 128)	SCA (*n* = 10)	*p*
Average heart rate, bpm	70 (62.2, 77.0)	77.5 (60.2, 98.0)	ns
SDNN, ms	87.0 (67.0, 106.7)	61.0 (53.5, 92.7)	.018
pNN50, %	4.0 (1.0, 11.0)	4.5 (1.5, 18.2)	ns
rMSSD, ms	23.5 (18.0, 36.0)	24.0 (17.0, 39.5)	ns
TP, ms^2^	1658.5 (1,077.7, 2,969.2)	882.5 (367.2, 1,375)	.007
VLF, ms^2^	1,173.5 (746.5, 2032.0)	453.5 (246.2, 999.2)	<.001
LF, ms^2^	239.5 (149.5, 437.5)	120.5 (53.2, 406.2)	ns
HF, ms^2^	130.5 (69.2, 279.7)	64.5 (34.7, 247.7)	ns
LF/HF	2.0 (1.0, 3.0)	1.0 (1.0, 3.2)	ns
QTc, ms	480.5 (453.2, 519.7)	523.5 (458.5, 560.2)	ns
TO	−0.9 (−1.7, 0.0)	0.0 (0.0, 1.1)	.004
TS, ms/RR	5.0 (2.0, 9.0)	2.0 (0.0, 4.1)	.009
VP ≥ 10/h, *n* (%)	30 (23.4)	5 (50.0)	ns
DC, ms	5.0 (4.0, 7.0)	3.0 (3.0, 5.0)	<.001
AC, ms	−5.8 (−7.2, −4.5)	−5.3 (−7.8, −3.4)	ns
DRs risk stratification			
Low‐risk, *n* (%)	79 (61.7)	0 (0.0)	<.001
Medium‐risk, *n* (%)	43 (33.6)	9 (90.0)	<.001
High‐risk, *n* (%)	6 (4.7)	1 (10.0)	ns

Abbreviations: AC, acceleration capacity of heart rate; DC, Deceleration capacity of heart rate; DRs, deceleration runs of heart rate; HF, high frequency; LF, low frequency; ns, no significance; SDNN, standard deviation of normal to normal RR intervals; VLF, very low frequency.

**Figure 1:**
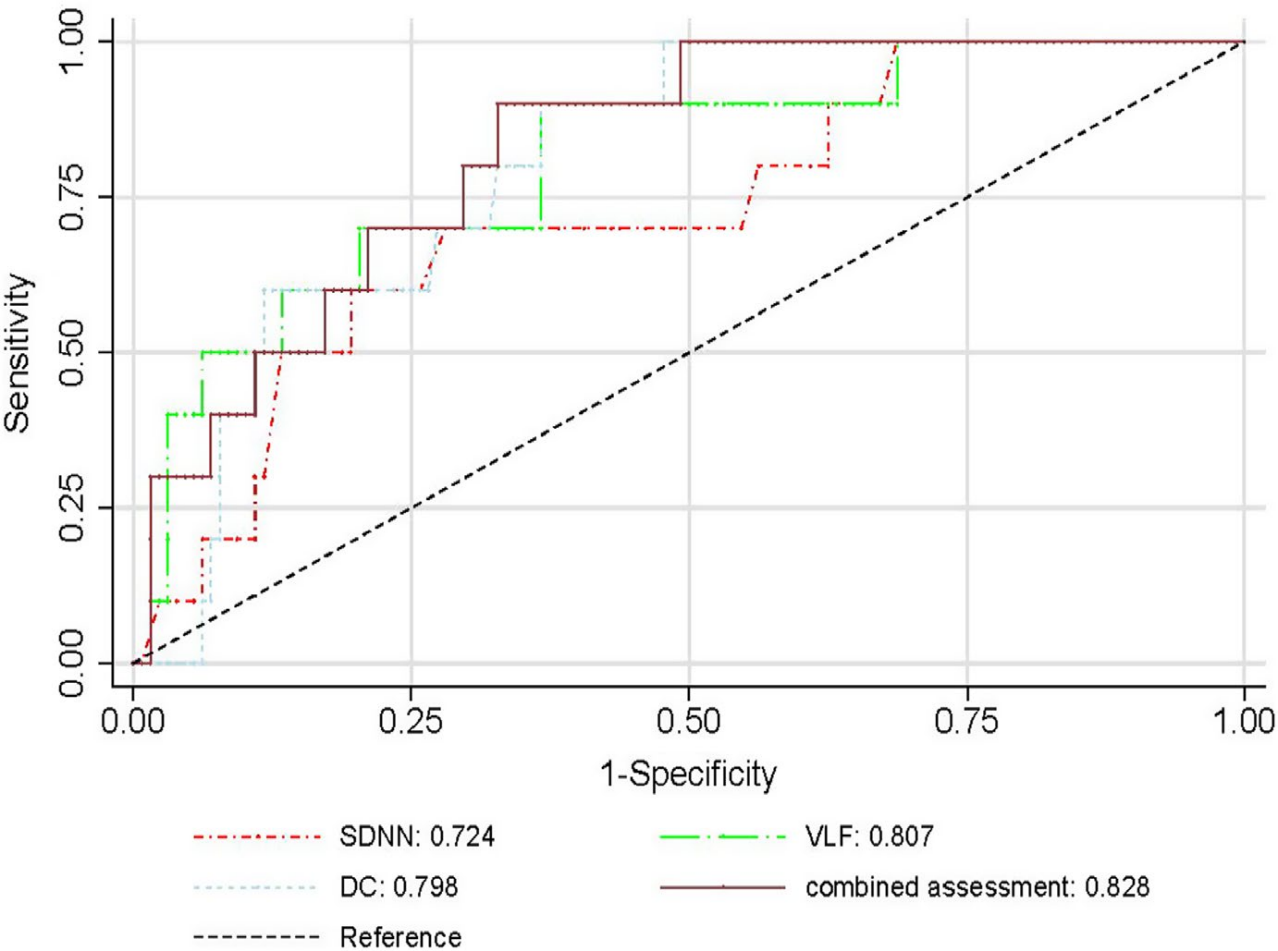
The AUC of VLF, DC, SDNN, and combined assessment for identifying SCA patients

On univariable regression, smoking, eGFR, SDNN, VLF, DC, and DRs risk stratification were risk factors of SCA, whereas the other indexes of heart rate variability showed no prognostic value. Dichotomous variables of SDNN, VLF, and DC, split by cut‐off values, were also incorporated into the competing‐risk regression separately. Presence of lower levels of SDNN, VLF, and DC was strong predictors of SCA, yielding hazard ratios of 5.5, 13.6, and 8.6, respectively. The results were shown in Table 3. The area under ROC curve (AUC) of SDNN, VLF, and DC for identification of SCA was 0.724 (*p* = .019), 0.807 (*p < *.001), 0.804 (*p* = .002), respectively. SDNN, VLF, and DC combined assessment area under ROC curve were 0.828 (*p < *.001) (Table 4, Figure 1).

**Table 3:** Univariable competing‐risk regression analysis for SCA

Variables	HR (95%CI)	*P*
Sex	0.444 (0.119–1.654)	ns
Age	1.078 (0.961–1.209)	ns
Smoking	0.230 (0.059–0.890)	.033
Hypertension	0.576 (0.168–1.973)	ns
Diabetes	0.729 (0.162–3.273)	ns
eGFR	0.977 (0.955–0.999)	.048
LVEF	0.972 (0.916–1.031)	ns
Grace score	1.018 (0.995–1.043)	ns
SDNN	0.967 (0.942–0.993)	.012
VLF	0.998 (0.996–0.999)	.027
DC	0.580 (0.445–0.754)	<0.001
SDNN^a^	5.545 (1.474–20.861)	.011
VLF^a^	13.650 (1.743–106.862)	.013
DC^a^	8.580 (2.410–30.551)	.001
DRs risk stratification	4.142 (2.554–6.717)	<.001

Abbreviations: DC, deceleration capacity of heart rate; DRs, deceleration runs of heart rate; eGFR, estimated glomerular filtration rate; ns, no significance; SDNN, standard deviation of normal to normal RR intervals; VLF, very low frequency.

^a^ Dichotomous variable: variables split by the cut‐off values.

**Table 4:** Predictive value of variables for SCA

Variables	AUC	95%*CI*	Cut‐off value	*P*	Sensitivity	Specificity
SDNN	0.724	0.570–0.878	69.5 ms	0.019	70.0%	71.9%
VLF	0.807	0.671–0.942	1,009.5 ms^2^	<0.001	90.0%	63.3%
DC	0.804	0.689–0.908	3.5 ms	0.002	60.0%	87.5%
Combined assessment	0.828	0.728–0.929	–	<0.001	90.0%	69.5%

Abbreviations: DC, deceleration capacity of heart rate; SDNN, standard deviation of normal to normal RR intervals; VLF, very low frequency.

**Figure 2:**
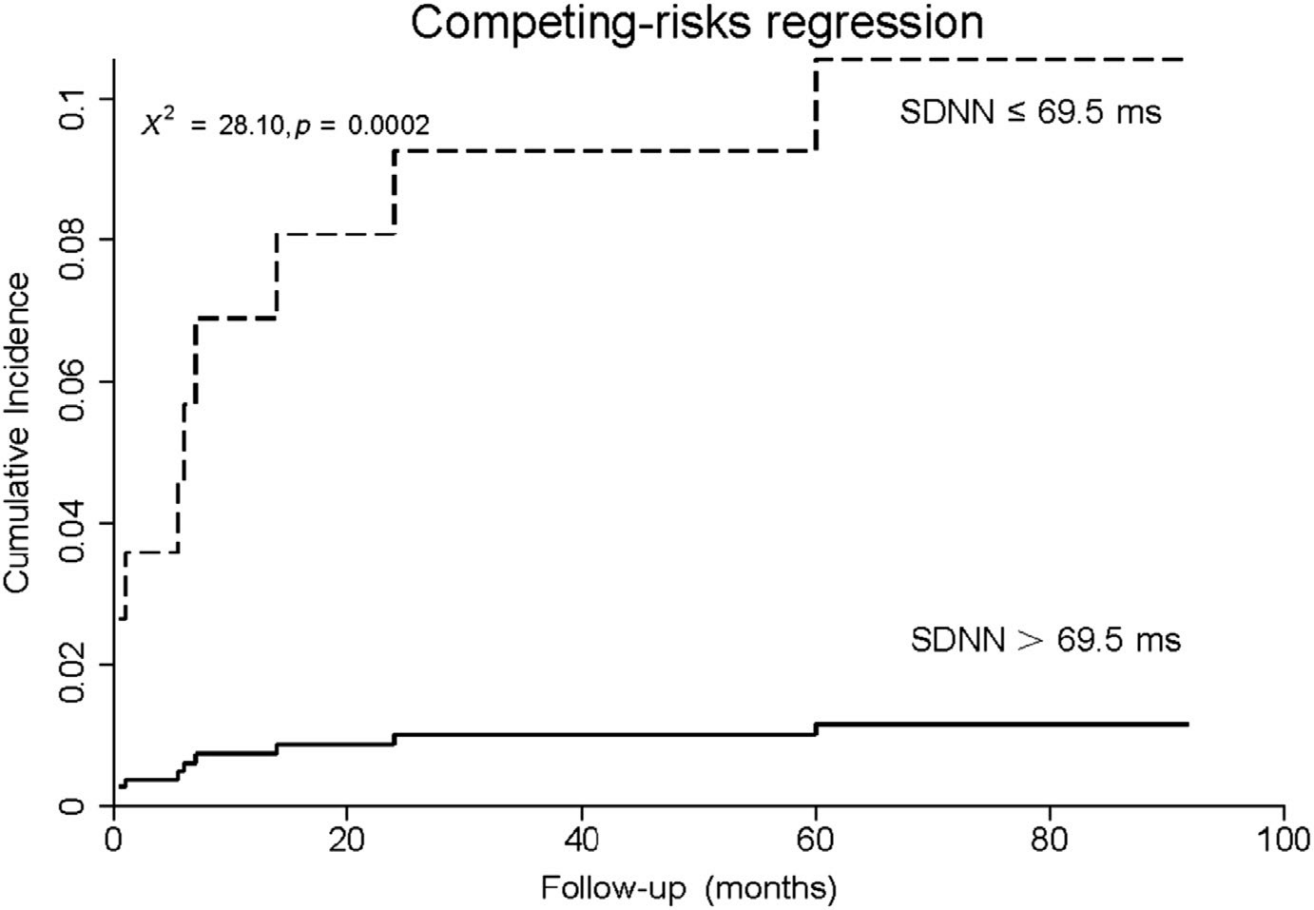
Cumulative incidence curve in AMI patients with LVEF ≥ 35% stratified by SDNN

**Figure 3:**
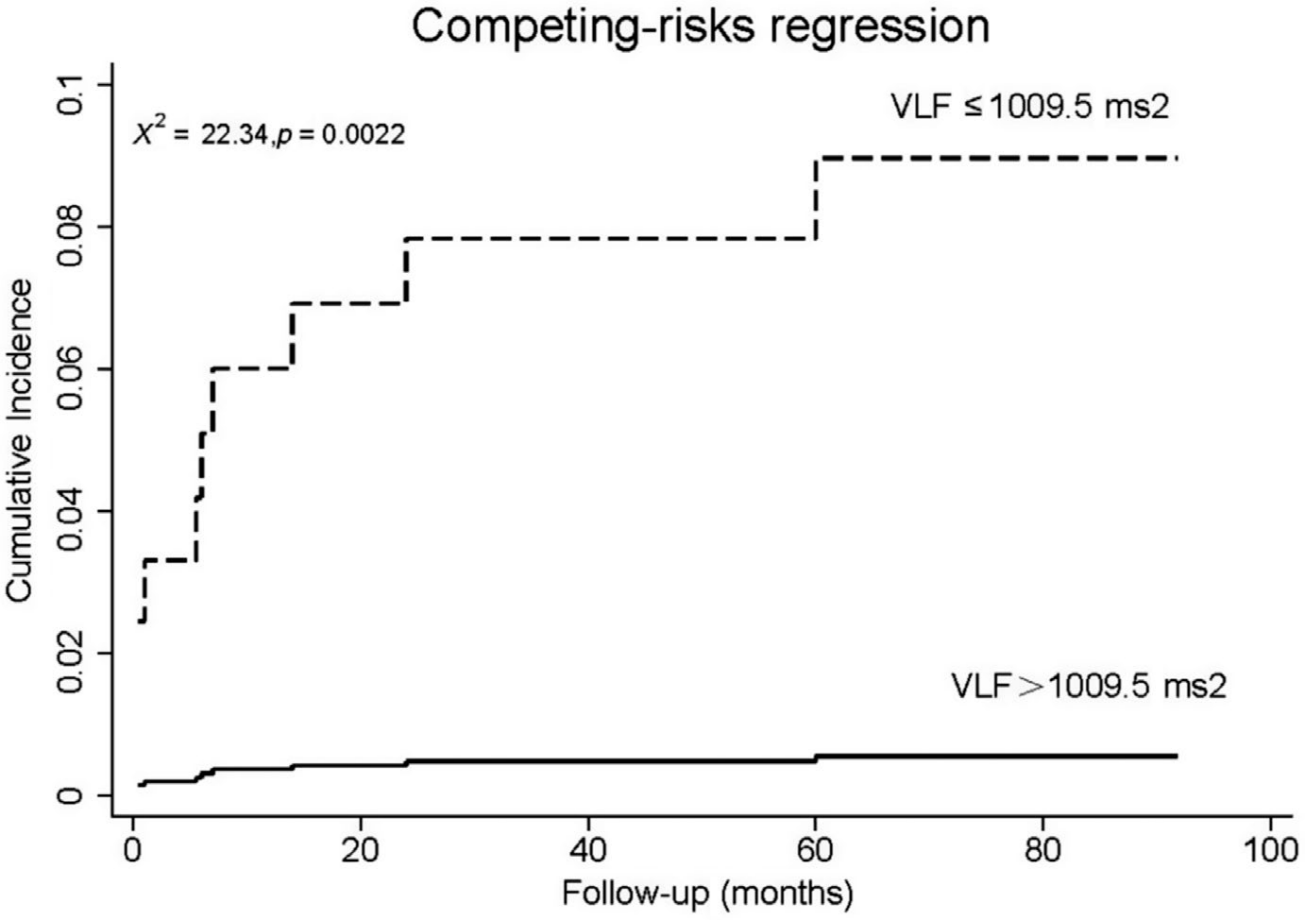
Cumulative incidence curve in AMI patients with LVEF ≥ 35% stratified by VLF

**Figure 4:**
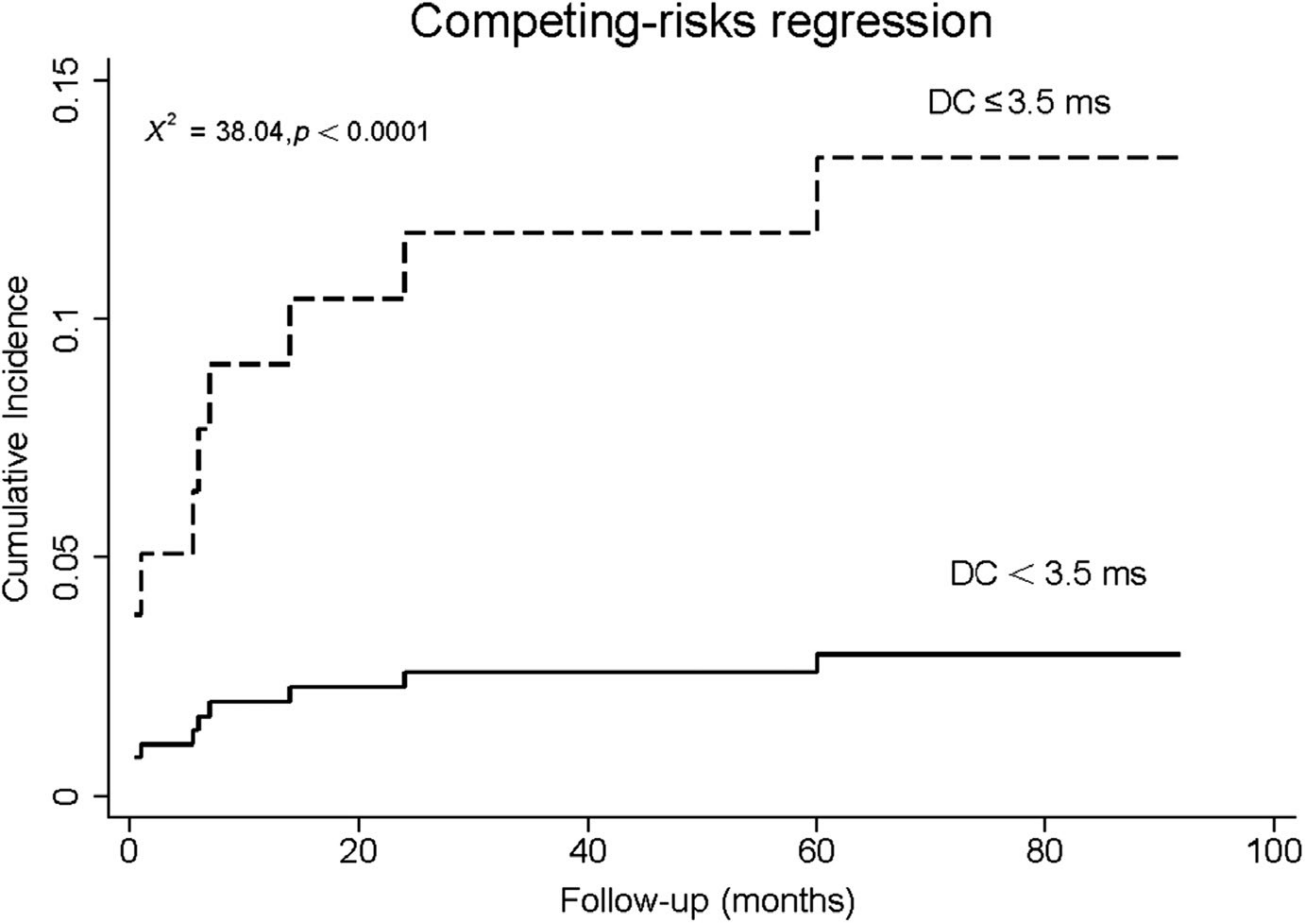
Cumulative incidence curve in AMI patients with LVEF ≥ 35% stratified by DC

Autonomic parameters were split into dichotomous variables pursuant to the cut‐off values separately. Multivariate competing‐risks regression incorporated one of the autonomic parameters, as well as standard risk factors into consideration scope, including variables with statistical significance in univariate model (smoking and eGFR), sex, age, diabetes, and LVEF. Split SDNN, VLF, and DC were evaluated separately. On multivariate competing‐risks regression, lower levels of SDNN, VLF, and DC remained significant predictors of SCA (Table 5), even after adjustment for traditional risk factors and other baseline covariates, yielding hazard ratios of 8.9, 14.7, and 4.4, respectively. The competing‐risks regression in Figures 2, 3, 4 showed obvious increases in cumulative incidence with the reduced levels of SDNN, VLF, and DC.

**Table 5:** Univariate and multivariate Cox regression analysis

	Unadjusted HR (95% CI)	*p*	Adjusted^b^ HR (95% CI)	*p*
Average heart rate	4.065 (1.147–14.411)	.030	—	ns
SDNN^a^	5.532 (1.430–21.402)	.013	8.888 (1.871–42.220)	.006
VLF^a^	14.060 (1.781–111.001)	.012	14.699 (1.653–130.691)	.016
DC^a^	8.589 (2.422–30.463)	<.001	4.430 (1.026–19.127)	.045
DRs stratification	4.143 (1.704–10.074)	.002	3.811 (1.060–13.706)	.040

^a^ Dichotomous variable.

^b^ hazard ratios adjusting for conventional risk factors (sex, age, smoking, diabetes, eGFR, and LVEF).

## 4 DISCUSSION

Our analysis from myocardial infarction patients with preserved or moderately reduced LVEF came to several findings. First, decreased SDNN, VLF, DC, and abnormal DRs were shown to be independently associated with increased risks of sudden cardiac arrhythmias in post‐MI patients with LVEF ≥ 35%, whereas other variables by time and frequency domain analysis of HRV did not have any prognostic values. Second, combined SDNN, VLF, and DC might help identify a high‐risk group of SCA.

Cardiac autonomic modulation dysfunction is involved in triggering of sudden cardiac death (La Rovere et al., 1998). The autonomic dysfunction in post‐MI patients usually exhibited sympathetic overactivity and loss of vagal tone (Hamm et al., 2017), in line with what we found in the present study. The loss of physiological function can be assessed by easily attainable holter‐related parameters such as diversified HR variability data, deceleration capacity, heart rate turbulence (turbulence onset and turbulence slope), and DRs. They were verified to be associated with long‐term mortality in previous studies (Bauer et al., 2006; Cygankiewicz, 2013; Deyell, Krahn, & Goldberger, 2015; Huikuri & Stein, 2013). In patients with myocardial infarction, these parameters have important clinical significance. For the past decades, numerous studies investigated the prognostic values of combined electrophysiological parameters, which were equally worse than that of patients with LVEF ≤ 35% (Bauer, 2017). From ISAR‐Risk study, DC and HRT identifies high‐risk post‐MI patients with LVEF > 30% for sudden cardiac death (SCD) (Bauer, Barthel, & Schneider, 2009), and combined periodic repolarization dynamics (PRD) and deceleration capacity helped identifies high‐risk postinfarction patients which might benefit from prophylactic strategies (Hamm et al., 2017). Severe autonomic failure (SAF), defined as abnormal heart rate turbulence with the presence of abnormal deceleration capacity (DC), was also proved to be associated with mortality risk (Bauer, Barthel, & Muller, 2009). A recent study introduced a formula combining noninvasive and invasive risk factors for risk stratification, and the two‐step algorithm yielded a sensitivity 100%, specificity 93.8%, which can be a guidance to ICD implantation (Gatzoulis, Tsiachris, & Arsenos, 2019). In the study we conducted, SDNN, VLF, and DC had been shown to be strong and independent predictors of SCA in myocardial infarction patients with preserved or moderately reduced LVEF. Combining the three parameters presented a prominent value of prognosis, implicating that the combined assessment of cardiac autonomic function utilizing SDNN, VLF, and DC might be a promising approach to identify high‐risk individuals after MI with LVEF ≥ 35%. Of course, studied variables combined with known risk factors may contribute significantly to the risk evaluation, and this deserves further study.

It should be noted that when comparing SCA and non‐SCA patients, SDNN, TP, VLF, DRs, and DC displayed remarkably statistical differences, whereas other autonomic data we selected lost significance. SDNN was one of the earliest and most widely used parameters for SCD prediction, and it was said to be independently associated with mortality and cardiac arrhythmias after myocardial infarction (Bigger et al., 1988; Kleiger, Miller, & Bigger, 1987; Lown & Verrier, 1976). In 1994, Sudhi et al. conducted a study about frequency domain analysis of HRV, in which the predictive value of rMSSD and pNN50 were lower than SDNN (Bigger et al., 1992), and rMSSD and pNN50 even showed no definite prognostic value according to another study (Vaishnav, Stevenson, & Marchant, 1994). Data on frequency domain analysis of HRV would be compromised by AMI events. Compared with the high‐frequency component, the low‐frequency component was significantly decreased in nonsurvival patients (Vaishnav et al., 1994). Bigger et al. found that LF and VLF had higher independent predictive power for all‐cause mortality after myocardial infarction (Bigger, Fleiss, Rolnitzky, & Steinman, 1993), and the study Farrell et al. conducted also suggested that LF was an effective predictor of arrhythmic events (SCD, nonsustained ventricular tachycardia, ventricular fibrillation) in postinfarction patients (Farrell, Bashir, & Cripps, 1991), whereas other frequency domain indices made no sense (Bigger et al., 1993). Also, the development of percutaneous transluminal coronary angioplasty (PTCA) and coronary artery bypass grafting (CABG) can diminish the associations between the HRV indices and mortality as reported before (Brateanu, 2015). Besides, the median age of SCA (79 years old) was higher than most previous studies (Bauer, Barthel, & Schneider, 2009; Bauer et al., 2006; Hamm et al., 2017). It is reported that HR variability lapses coming with age, and values of representative indices of the parasympathetic nervous system were lower in older persons (Spina, Gonze, & Barbosa, 2019; Voss, Schroeder, & Heitmann, 2015), which might diminish the differences between SCA and non‐SCA. Furthermore, the system of heart rate regulation seems to be multilevel and complicated, factors such as gender, drug interferences, and concomitant diseases might also be involved. The present study was consistent with the previous research results, showing the superiority of SDNN and VLF in risk prediction for SCD than other indexes of HRV.

Another novel founding of our study was the great prognostic value of DRs than other conventional autonomic parameters. DRs, short for heart rate deceleration runs, constructed one part of the heart rate asymmetry and were a method based on monotonic runs of heart rate decelerations. Separated from complex patterns of continuous RR interval time series, DRs quantifies the heart rate deceleration based on simple counts of gradually prolonging RR intervals (Guzik et al., 2012; Jiang, Chen, & Zhang, 2017). It reflexes the ability of predominantly vague nerves to slow heart rate beyond a single beat‐to‐beat change (Guzik et al., 2012). As a method implementing the concept of deceleration capacity, DRs were divided into low‐, intermediate‐, and high‐risk groups in past studies, and quantitatively evaluate the modulation of vagus nervous on the sinus node. However, DRs have disappeared in cardiologists’ view as an independent marker for prognosis in recent years to the extent of our knowledge. One study by Guzik P et al. showed that infrequent DRs are powerful indicators of a high risk of postinfarction mortality, and the 2‐year total mortality probabilities gradually increased for low‐, intermediate‐, and high‐risk groups, and the differences between the high‐risk group and the 2 other groups were highly different (Guzik et al., 2012). The findings were partly in agreement with the results of competing‐risks regression analysis of our data, and verified the reliability and usefulness of DRs in the prediction of sudden cardiac events in postinfarction patients with LVEF ≥ 35%.

The study conducted by us has certain limitations. First, what we must recognize is that the conclusions we have are based on a limited simple size with a large time span and a low number of endpoints. It is probably more appropriate to interpret the findings of our study as hypotheses. It still remains unknown how external conditions such as introduced new drugs in recent years correlate with the long‐term arrhythmia and our results. The hypothesis‐generating results we came to need further verification in a larger study in the future. Second, we did not take heart rate turbulence into consideration for the limitation of a lower simple size. Furthermore, subjects enrolled were the elderly with a median age 65 years old. Thus, it is uncertain whether the conclusions can be used in younger ones. However, despite these limitations, the results of our study can provide ideas and methods for risk prediction for sudden cardiac death in postinfarction patients with LVEF ≥ 35%.
